# Source genotype influence on cross species transmission of transmissible spongiform encephalopathies evaluated by RT-QuIC

**DOI:** 10.1371/journal.pone.0209106

**Published:** 2018-12-20

**Authors:** Soyoun Hwang, Justin J. Greenlee, Natalie M. Vance, Eric M. Nicholson

**Affiliations:** United States Department of Agriculture, Agricultural Research Service, National Animal Disease Center, Virus and Prion Research Unit, Ames, Iowa, United States of America; Creighton University, UNITED STATES

## Abstract

Scrapie is a naturally occurring transmissible spongiform encephalopathy of sheep and goats. This fatal neurodegenerative disease is caused by misfolding of the cellular prion protein to pathogenic β-rich conformers (PrP^Sc^) that accumulate in higher order structures of the brain and other tissues. This conversion has been used for *in vitro* assays including serial protein misfolding amplification and real-time quaking induced conversion (RT-QuIC). RT-QuIC can be used for the detection of prions and for strain discrimination in a variety of biological tissues from humans and animals. In this study, we evaluated how PrP^Sc^ isolated from sheep of different genotypes after inoculation with the scrapie agent influence the fibril formation *in vitro* using RT-QuIC. We found that reaction mixtures seeded with PrP^Sc^ from genotype VRQ/VRQ sheep brains have better conversion efficiency with 132M elk substrate compared to reactions seeded with PrP^Sc^ from the brains of sheep with the ARQ/ARQ genotype no matter which strain of scrapie was used to seed the reactions. We also inoculated transgenic mice expressing 132M elk *PRNP* (Tg12) with the scrapie agent from different genotypes of sheep to compare with our RT-QuIC results. The bioassays support the data showing a significantly shorter incubation period for inoculum from VRQ/VRQ sheep when compared to inoculum from ARQ/ARQ sheep. Thus, we conclude that the genotype of both source and recipient can strongly influence transmission.

## Introduction

Prion diseases are a group of fatal neurologic diseases that result from the misfolding of the cellular prion protein (PrP^C^) into a pathogenic form (PrP^Sc^) primarily in the brain and spinal cord. These diseases also are known as transmissible spongiform encephalopathies (TSEs). Prion diseases include scrapie in sheep, chronic wasting disease (CWD) in cervids, bovine spongiform encephalopathy (BSE) in cattle and Creutzfeldt-Jakob disease (CJD), fatal familial insomnia (FFI), Gerstmann-Sträussler-Scheinker syndrome (GSS), and kuru in humans. Misfolded proteins accumulate in the central nervous system in all TSEs, and in cases of scrapie in sheep and CWD in cervids, PrP^Sc^ also accumulates in the lymphoid tissues [1–4].

TSE agents exist as different strains. Rodent bioassays can be considered a definitive technique for the recognition and characterization of different prion strains and are a common approach to assessing the host range of a TSE isolate in instances when use of the natural host species is not feasible. However, biochemical approaches also can be applied to assess the different PrP^Sc^ types including comparison of migration patterns of proteinase K (PK) resistant PrP^Sc^ glycoforms by western blotting. Alternatively, strains can be characterized with regard to fibril stability by unfolding PrP^Sc^ using guanidine hydrochloride (GdnHCl) with fibril unfolding monitored by western blot [5] or enzyme immunoassay (EIA/ELISA) [6]. Real time quaking induced conversion (RT-QuIC) is an efficient and sensitive tool to detect prions in various samples from humans and animals. In addition, this technique has shown potential analytical applications such as prion strain discrimination, drug screening, and screening for prion contamination [7]. Recently, differences in amplification using RT-QuIC were shown to discriminate prion strains [8–14].

Two distinct strains of US sheep scrapie isolates, No. 13–7 and x124, have been investigated in several studies [6, 15–19]. The No. 13–7 agent was passaged in 4 generations of sheep and was shown to be stabilized in Suffolk sheep [15]. No. 13–7 inoculum induced scrapie within an average of 19 months when transmitted by intracerebral inoculations, while x124 inoculum induced scrapie much faster. Sheep inoculated with these two inocula showed different clinical signs, incubation times, spongiform lesion profiles, genotypes of susceptible sheep and fibril stabilities [6, 18, 19] with the ultimate conclusion that they are two distinct strains [19].

PrP polymorphisms influence prion pathogenesis in human and animals. In sheep, the relative susceptibility to classical scrapie infection is heavily influenced by polymorphisms occurring at residues 136 [valine (V) or alanine (A)], 154 [arginine (R) or histidine (H)] and 171 [glutamine (Q), R, H or lysine (K)]. Sheep homozygous for the VRQ allele have increased susceptibility, whereas sheep homozygous for the ARR allele are resistant to sheep scrapie [20–23]. In elk, the primary susceptibility/resistance associated polymorphism is at residue 132 with methionine (M) associated with higher CWD susceptibility and leucine (L) associated with higher resistance. Experimental oral challenge of elk indicated that the susceptibility/resistance conferred at position 132 is relative and that MM132 homozygous elk had the shortest incubation period. Heterozygous LM132 elk had longer incubation periods nearly double that of MM132, and elk with the LL132 genotype had incubation periods that were three times longer than elk with the MM132 genotype [24–26]. In the analogous human scenario, all documented clinical cases of vCJD have occurred in patients with at least one methionine at codon 129 in *PRNP* sequence (MM or MV) [27–30].

Elk are susceptible to the sheep scrapie agent when inoculated intracerebrally. When challenged with the sheep scrapie agent, elk with MM at residue 132 have a shorter incubation period, and elk with the heterozygous (LM) genotype have a longer incubation period [31]. However, the susceptibility of elk of different *PRNP* genotypes to scrapie isolates from sheep of different *PRNP* genotypes remains unknown. Therefore, investigating how different genotypes of sheep scrapie affect the transmission to cervid hosts with differing prion protein polymorphisms may help us to further understand the risks of transmission of scrapie to cervids and may also provide important information with regard to understanding the origin of CWD. In this study, we utilized the *in vitro* assay, RT-QuIC, to evaluate how the *PRNP* genotype of the infectious seed influences the *in vitro* conversion using sheep scrapie agent of different genotypes and strains as a seed for RT-QuIC based conversion of 132M and 132L elk prion protein. This assessment will determine if different strains or different genotypes of PrP^Sc^ present in sheep scrapie may afford a higher risk of transmission to cervids. Where possible, these results were compared to mouse bioassay transmission of the scrapie agent from different genotypes of sheep to transgenic mice expressing elk prion protein.

## Materials and methods

### Ethics statement

All animal experiments described were reviewed and approved by the National Animal Disease Center’s Institutional Animal Care and Use Committee (protocol numbers: ARS-2017-629). The animal experiments were carried out in accordance with the Guide for the Care and Use of Laboratory Animals (Institute of Laboratory Animal Resources, National Academy of Sciences, Washington, DC). Details concerning procedures are included under the section Mouse Bioassay.

### Sources of inocula and RT-QuIC seed

Brain samples from scrapie-infected sheep were obtained from studies previously conducted at the National Animal Disease Center. Briefly, lambs were inoculated with either x124-infected samples intracerebrally (IC) [19] or 13–7 infected samples intranasally (IN) [15] After inoculation the animals were allowed to progress to incidence of clinical disease, euthanized, and brain samples collected and stored at -80°C prior to use. The classification of these isolates as different strains of scrapie using a combination of biochemical and pathologic approaches is well defined [6, 19]. One non-inoculated sheep that did not develop clinical symptoms during the time frame of the experiment was used as a negative control. Animals, breeds, genotypes, inoculum source and time to disease on set are summarized in Table 1.

**Table 1:** Clinical information for sheep samples.

Animal	Breed	Genotype	Inoculum	Incubation period (mo.)	Ear tag #	EIA (OD)
1	Cheviot	ARQ/ARQ	X124	16.5	709	4.0
2	Cheviot	ARQ/ARQ	X124	16	722	4.0
3	Cheviot	VRQ/VRQ	X124	5	720	4.0
4	Cheviot	VRQ/VRQ	X124	4.5	744	4.0
5	Suffolk	VRQ/VRQ	No. 13–7	26	813	4.0
6	Suffolk	VRQ/VRQ	No. 13–7	27	814	4.0
7	Suffolk	ARQ/ARQ	No. 13–7	21	822	4.0
8	Suffolk	ARQ/ARQ	No. 13–7	17	823	4.0
9	Suffolk	ARQ/ARQ	Neg-control	End of study	828	0.07

### Western blotting of brain homogenates from sheep

Samples of sheep brainstem that had been frozen at -80°C were used to prepare brain homogenate for western blotting. Brain samples were bead homogenized at 20% (w/v) in 1X PBS (Dulbecco’s PBS, pH 7.4, lacking calcium and magnesium) and tested shortly after storage at -80°C. PK digestion of whole brain homogenate was performed in 1X PBS for 1 hour at 37°C, at a final PK of 50 μg/ml, and then each sample was separated by SDS-PAGE on 12% polyacrylamide minigels (Invitrogen) and then transferred onto polyvinylidene difluoride (PVDF) membranes (Millipore, Billerica, MA) for 45 min at 30 V. The membranes were blocked with 3% bovine serum albumin (BSA) in Tris-buffered saline (TBS) for 1 h and incubated at 4° C overnight with mouse anti-PrP monoclonal antibody P4 at a 1:10,000 dilution (0.1 μg/ml) as the primary antibody. Then, a biotinylated sheep anti-mouse secondary antibody at 0.05 μg/ml and a streptavidin–horseradish peroxidase (HRP) conjugate were used in conjunction with a chemiluminescent detection system (Pierce ECL plus, Thermo Fisher) and visualized on an imaging system capable of detecting luminescence.

### Enzyme immunoassay (EIA) of brain homogenates from sheep

The IDEXX HerdChek BSE-Scrapie Antigen EIA test kit was used to selectively detect the presence of disease associated misfolded prion protein in sheep brains. Brain homogenates from scrapie-infected sheep were assessed using the IDEXX HerdChek BSE EIA kit in the absence of proteinase K digestion. EIA was performed as described by the manufacturer. The cutoff value was determined by the negative control sample provided by the manufacturer and the optical density value was around 0.07 ± 0.005. If the optical density value was over 0.15, the samples were considered positive. All brain samples were normalized with EIA kit before analysis by RT-QuIC by diluting to an O.D. around 1.0.

### Recombinant prion protein production and purification

*E*. *coli* (BL21(λDE3)) was transformed with the pET28a vector containing the wild type elk PrP gene (amino acids 26–234, equivalent to human 23–231; GenBank accession number AAC12860.2) and the recombinant elk prion proteins were expressed and purified as described by Vrentas *et al* [32]. The concentration of pooled protein eluent was measured by UV and calculated from the absorbance at 280 nm using an extinction coefficient of 59,485 M^-1^cm^-1^ as calculated for mature elk proteins (23–231), 132M and 132L proteins.

### RT-QuIC protocol

RT-QuIC reactions were performed as previously described [9–12, 33–35]. The reaction mix was composed of 10 mM phosphate buffer (pH 7.4), 100 mM to 500 mM NaCl, 0.1 mg/ml recombinant elk prion proteins 132L or 132M, 10 μM thioflavin T (ThT), and 1 mM ethylenediaminetetraacetic acid tetrasodium salt (EDTA). Aliquots of the reaction mix (98 μL) were loaded into each well of a black 96-well plate with a clear bottom (Nunc, Thermo Fisher Scientific) and seeded with 2 μL of brain homogenate dilutions. The plate was then sealed with plate sealer film and incubated at 42°C in a BMG FLUOstar Omega plate reader with cycles of 15 min shaking (700 rpm double orbital) and 15 min rest for 100 h. ThT fluorescence measurements (excitation, 460 nm; emission 480 nm, bottom read, 20 flashes per well, manual gain 1400) were taken every 45 min.

To optimize the conditions of the RT-QuIC reaction, assays using brain samples from negative control or scrapie-infected sheep were performed with 132M and 132L substrates with or without SDS and at different NaCl concentrations (100, 200, 300, 400, 500 mM). When optimized, RT-QuIC was performed using quadruplicates of two dilutions (10^−2^ or 10^−3^) of non-inoculated control or infected sheep brain homogenates (animal #2, #3, #5 and #7). All reactions for each dilution and each sample were performed twice with each individual experiment each consisting of 4 technical replicates for a total of 8 replicates per sample. ThT fluorescence data are displayed as the average ThT fluorescence for an individual experiment consisting of four technical replicates for each time point and, to be considered positive, the ThT fluorescence of at least two out of four replicates must be positive. As previously described for classification of positive samples by RT-QuIC, the positive threshold was calculated as the mean value of non-inoculated control sheep brain homogenates plus 10 standard deviations and the time to the positive threshold is referred to as the lag time [33, 36, 37].

### Mouse bioassay

Mice expressing the elk prion protein (Tg(ElkPrP-132M)Prnp^0/0^ mice; Tg12) [38] were used to compare the incubation periods and attack rates of scrapie isolates used for RT-QuIC. Mice (n = 24 for x124 inoculum from a VRQ/VRQ sheep; n = 25 for No. 13–7 inoculum from an ARQ/ARQ sheep) were inoculated intracerebrally under isoflurane anesthesia as previously described [39] using 20 μl of a 1% (wt./vol.) brain homogenate from sheep with clinical signs of scrapie. Mice were housed in individually ventilated cages with ad libidum access to food and water in a room with a 12 hour light/dark cycle. Mice were monitored daily for the development of clinical signs suggestive of prion disease such as ataxia, listing or rolling gate, pelvic limb paresis, lethargy, or poor grooming with urine stained fur by animal care staff. Mice were humanely euthanized in a CO_2_ chamber by trained personnel if the aforementioned signs suggestive of prion disease were observed or at the end of the experiment (700 days post-inoculation) and tissues were collected for analysis. Positive/negative status of individual mice was determined by EIA (see above). Survival analysis was done using Graph Pad Prism 7 (GraphPad Software, San Diego) with incubation periods shown in days post-inoculation. In total 10 mice were found dead without observation of clinical signs. Three of those had high levels of detectable PrP^Sc^ based upon EIA assay and were likely the result of rapid onset of clinical signs. The remainder died from concurrent disease issues (neoplasms) due to the long term nature of the experiment.

## Results

### Western blotting and quantitation of PrP^Sc^ by EIA in brain samples from negative control and scrapie-infected sheep

**Fig 1:**
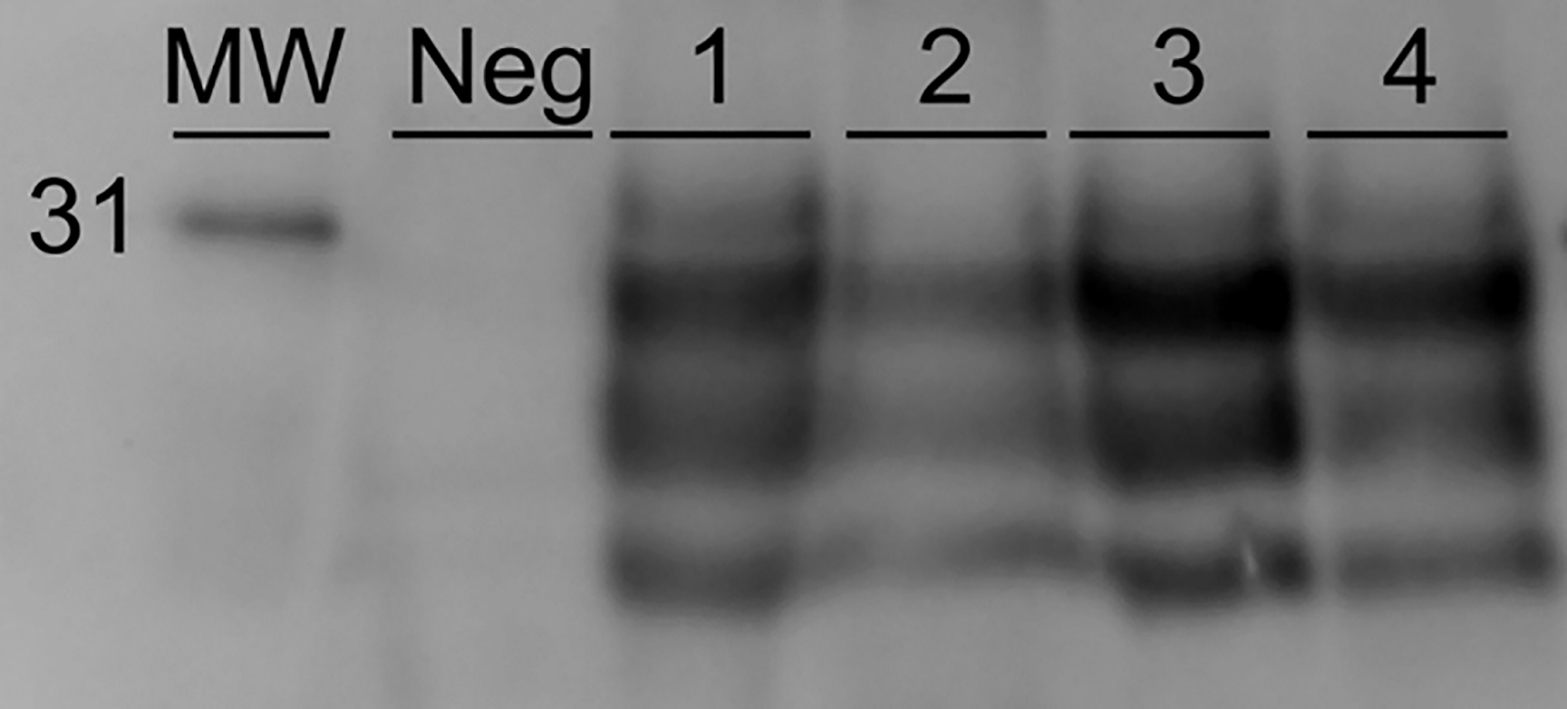
Western blot patterns of representative PrP^Sc^ from x124 infected and No. 13–7 infected sheep brains. Each lane represents PK-digested PrP^Sc^ probed with an anti-PrP monoclonal antibody P4. Lane key: MW, molecular weight marker. Neg depicts normal sheep brain. Lane 1, animal 1, ARQ/ARQ sheep challenged intracerebrally with x124. Lane 2, animal 3, VRQ/VRQ sheep challenged intracerebrally with No. x124. Lane 3, animal 5, VRQ/VRQ sheep challenged intranasally with No. 13–7. Lane 4, animal 7, ARQ/ARQ sheep challenged intranasally with No. 13–7.

Brainstem samples (obex) were collected from sheep with clinical signs of scrapie. To confirm that strain specific properties of each inoculum were transmitted to the experimental animals, brainstem samples from selected animals were characterized by western blot using the P4 antibody (Fig 1). These brain samples were sourced from previously published studies [6, 18, 19], so only a representative brain sample of each sheep *PRNP* genotype was tested. Molecular profiles obtained from western blot of brain homogenates from representative sheep were similar regardless of inoculum used or genotype of sheep or route of inoculation in a comprehensive analysis of these isolates [19].

EIA was performed on brainstem samples from all scrapie-inoculated sheep to determine the relative amount of misfolded prion protein. High levels of PrP^Sc^ were detected in the brainstem of sheep with clinical signs, but not in samples from the negative controls (Table 1).

### Optimization of RT-QuIC for the discrimination of scrapie prion seeding activity

**Fig 2:**
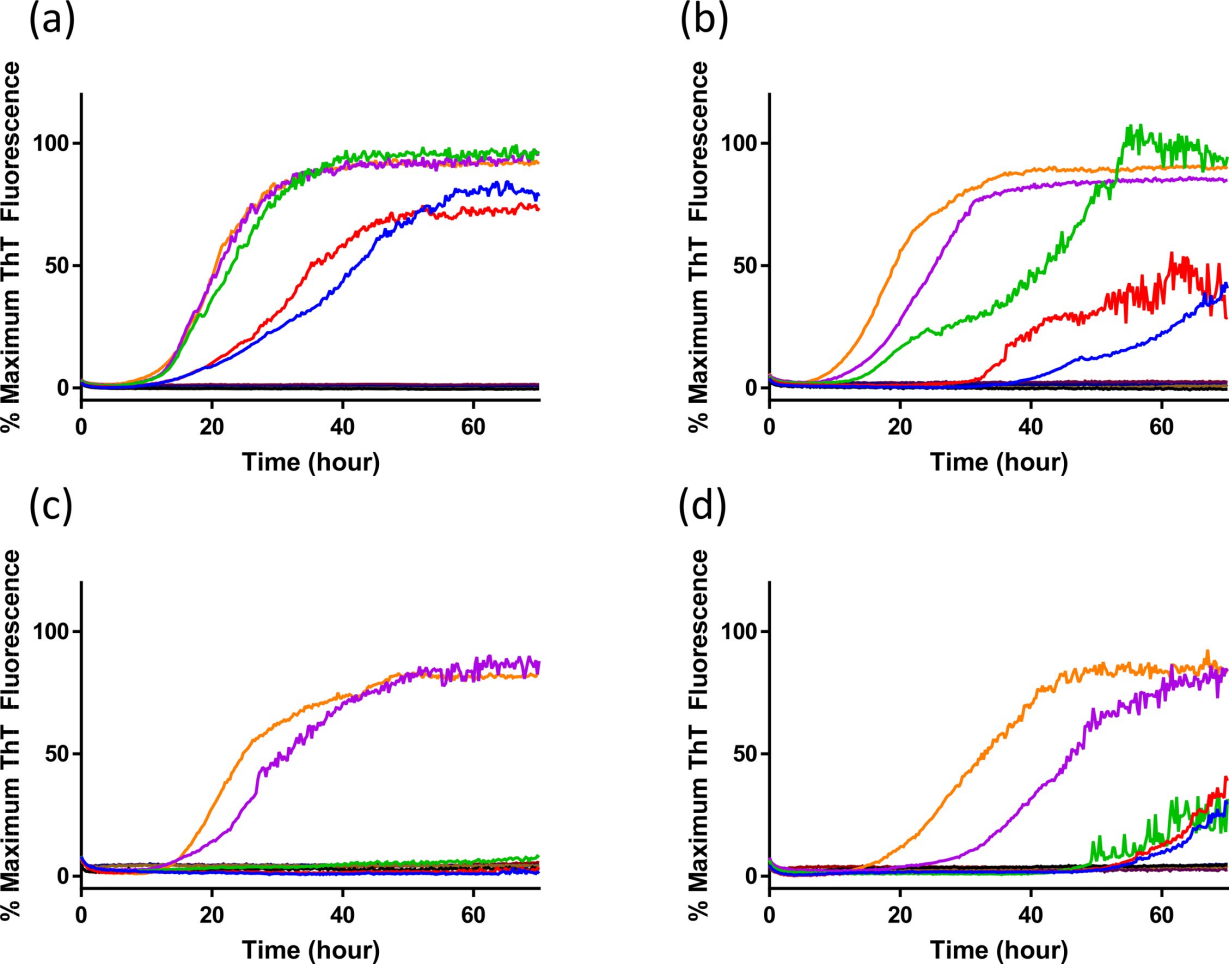
RT-QuIC sodium chloride titration for scrapie-infected and uninfected sheep brain samples. RT-QuIC reactions were seeded with 10^−2^ brain homogenate of animal number 3 (VRQ/VRQ;x124) using full-length elk prion protein 132L (a) and 132M (b) using a range of NaCl concentrations (100–500 mM, 100mM: blue, 200mM: red, 300mM:green, 400mM: purple, 500mM: orange line). Each reaction was seeded with 10^−3^ brain dilution using full-length elk prion protein 132L (c) and 132M (d). Data are presented as mean ThT fluorescence of 4 technical replicates. Uninfected sheep brain samples showed no increase in ThT regardless of NaCl concentration and the individual curves are not distinguishable as individual curves and appear as a thick dark line near 0% Maximum ThT Fluorescence.

**Fig 3:**
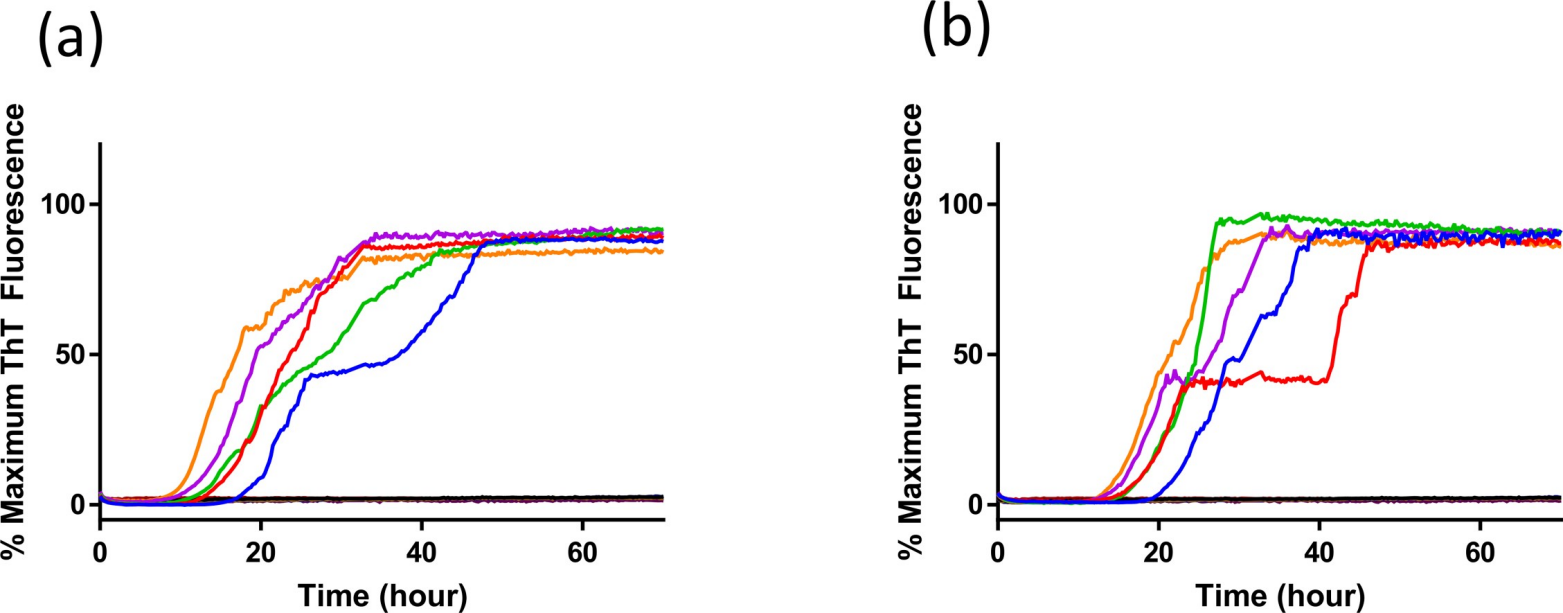
RT-QuIC sodium chloride titration for scrapie-infected and uninfected sheep brain samples. RT-QuIC reactions were seeded with 10^−2^ brain homogenate of animal number 2 (ARQ/ARQ;x124) using full-length elk prion protein 132L (a) and 132M (b) using a range of NaCl concentrations (100–500 mM, 100 mM: blue, 200 mM: red, 300 mM:green, 400 mM: purple, 500 mM: orange line). Data are presented as mean ThT fluorescence of 4 technical replicates. Uninfected sheep brain samples showed no increase in ThT regardless of NaCl concentration and the individual curves are not distinguishable as individual curves and appear as a thick dark line near 0% Maximum ThT Fluorescence.

Use of RT-QuIC for diagnostic purpose necessitates optimal sensitivity and for each seed and substrate combination a set of optimal solution conditions is determined. However, for discrimination of seeding a single condition set must be selected that is suitable for all seed and substrate combinations. To accomplish this we first compared the effects of NaCl concentration with the different elk PrP substrates for RT-QuIC amplification of x124 inoculated VRQ/VRQ sheep (Fig 2) and ARQ/ARQ sheep (Fig 3) sheep scrapie. As can be seen in Fig 2A and 2B, assays with reaction mixture containing from 300 mM to 500 mM NaCl with 132L and 132M substrates show reduced lag time and higher amount of fibril formation compared to reaction mixtures containing 100 mM and 200 mM of NaCl when they are seeded with brain sample from animal #3 (VRQ/VRQ scrapie-infected sheep). Also, 400 mM and 500 mM NaCl exhibited higher sensitivity with both substrates by showing positive seeding activities of 132L (Fig 2C) and 132M (Fig 2D) substrates for 10^−3^ brain dilution. Reactions seeded with brain homogenate from scrapie-infected sheep in the presence of higher NaCl concentrations show ThT fluorescence indicating higher conversion efficiency for both substrates. No increase in ThT fluorescence is observed when normal brain homogenate is used as the seed at any of the NaCl concentrations evaluated in this study. When using lower NaCl concentrations (100 mM and 200 mM NaCl), there is increased ThT fluorescence for both substrates with a longer lag time with lower amounts of fibril formation without spontaneous fibril formation. Optimization with seed from different genotypes of sheep (ARQ/ARQ) was also done with each substrate (132L (Fig 3A) and 132M (Fig 3B)). Compared to the assays seeded with brain from VRQ/VRQ sheep, seeding activity is less dependent on NaCl concentrations.

**Fig 4:**
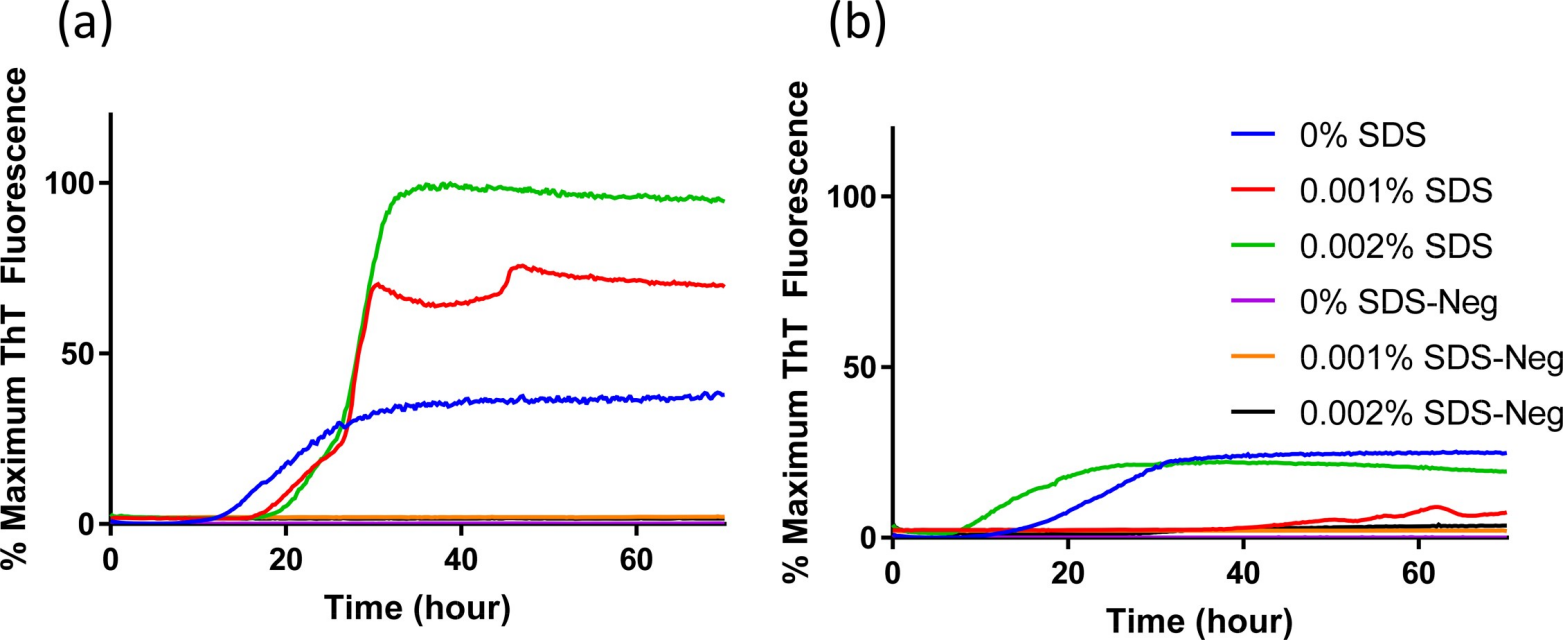
**Comparison of SDS dependency of RT-QuIC reactions using mature (a) elk prion protein 132L or (b) 132M as substrates.** RT-QuIC reactions were seeded with 10^−2^ dilutions of scrapie-infected sheep (animal #3) and negative control (uninfected sheep) brain homogenates using either mature elk prion protein 132L or 132M protein (aa 23–231) as substrates without SDS (blue line), with the addition of 0.001% (red line) or 0.002% SDS (green line) in the presence of 400 mM NaCl. Data are presented as mean ThT fluorescence of 4 technical replicates.

The impact of SDS on the seeding activity with scrapie prion was also tested (Fig 4A and 4B). For 132L substrate, assays exhibited little SDS dependence to lag time (Fig 4A). For assays conducted in 132M substrate, the presence of 0.002% SDS reduced the lag time for prion seeding activity by 10 h compared to assays without SDS (Fig 4B). Interestingly, a concentration of 0.001% SDS did not support RT-QuIC for the 132M substrate based on the absence of any increase in fluorescence. Under no conditions investigated did the negative control sample show conversion in the RT-QuIC assay.

**Fig 5:**
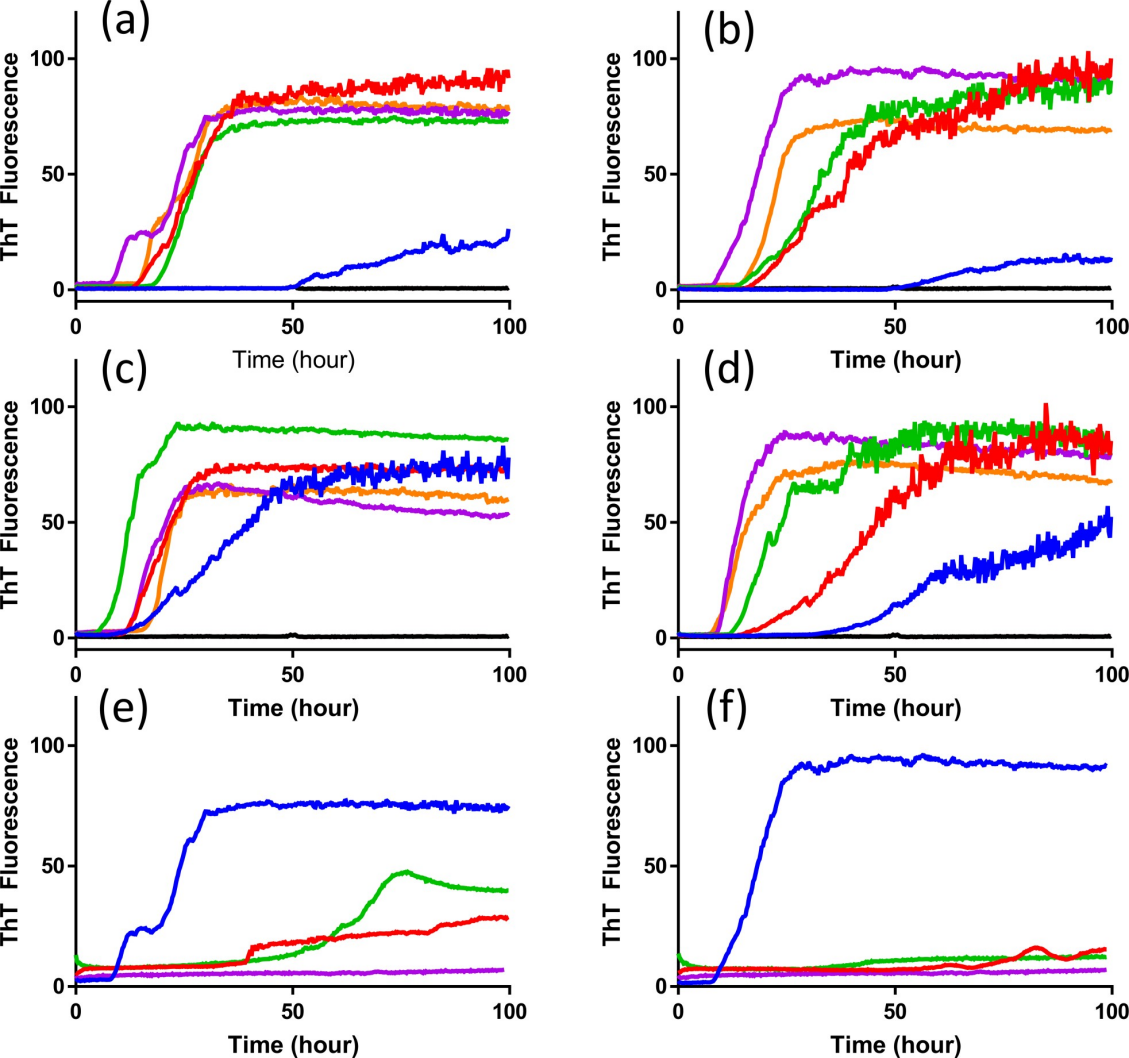
RT-QuIC sodium chloride and SDS titration for 13–7 scrapie-infected and uninfected sheep brain samples. RT-QuIC reactions were seeded with 10^−2^ brain homogenate of animal number 5 (VRQ/VRQ;13–7) and 7 (ARQ/ARQ; 13–7) using full-length elk prion protein 132L (a and c) and 132M (b and d) using a range of NaCl concentrations (100–500 mM, 100 mM: blue, 200 mM: red, 300 mM:green, 400 mM: purple, 500 mM: orange line). Uninfected sheep brain samples showed no increase in ThT regardless of NaCl concentration and the individual curves are not distinguishable as individual curves and appear as a thick dark line near 0% Maximum ThT Fluorescence (a, b, c, and d). RT-QuIC reactions were also seeded with 10^−2^ dilutions of 13–7 scrapie-infected sheep (animal #5) and negative control (uninfected sheep) brain homogenates using either mature elk prion protein 132L (e) or 132M (f) protein (aa 23–231) as substrates without SDS (blue line), with the addition of 0.001% (red line) or 0.002% SDS (green line) in the presence of 400 mM NaCl. Negative control is shown in purple for both substrates with 0% SDS and 400 mM NaCl. Data are presented as mean ThT fluorescence of 4 technical replicates.

Reaction conditions containing 300 mM to 500 mM NaCl and either 0% or 0.002% SDS were determined to be equivalently optimal for x124 scrapie detection when using RT-QuIC assays with recombinant elk prion proteins. For 13–7 scrapie the range extended from 200 mM to 500 mM NaCl, and 0% SDS was found to support better conversion for both substrates by RT-QuIC (Fig 5). Thus the optimal conditions supporting conditions for both scrapie isolates was found to be 400 mM NaCl and 0% SDS.

### Genotype influence on seeded conversion of elk PrP

**Fig 6:**
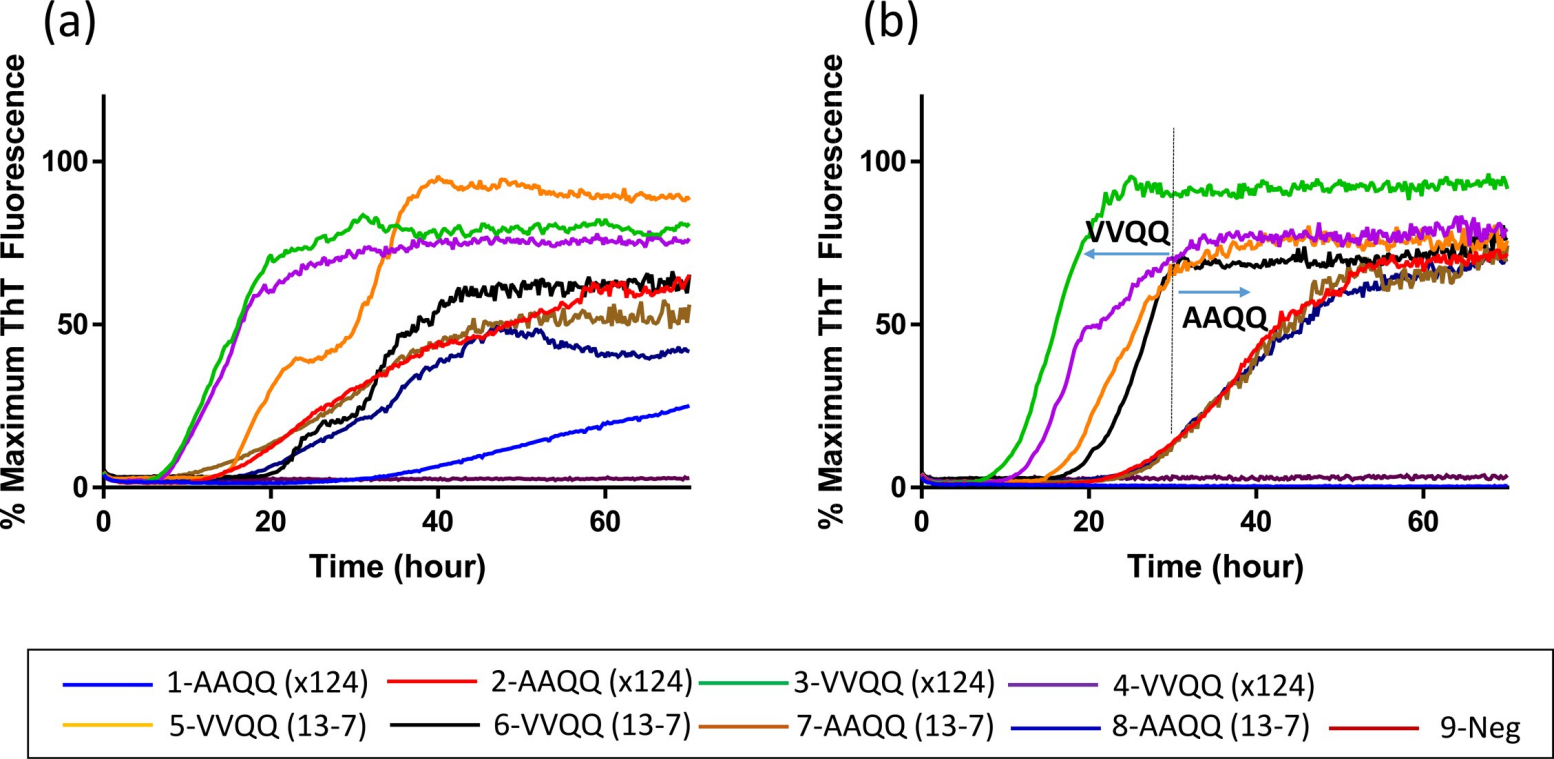
**RT-QuIC detection of seeding activity of scrapie prions from sheep brain samples using mature elk prion protein 132L (a) and 132M (b) (23–231).** RT-QuIC reaction mixtures were seeded with 10^−2^ of EIA normalized brain tissues from uninfected, x124 infected sheep. A final 400 mM NaCl was used with the substrates. Data are presented as mean ThT fluorescence of 4 technical replicates.

**Fig 7:**
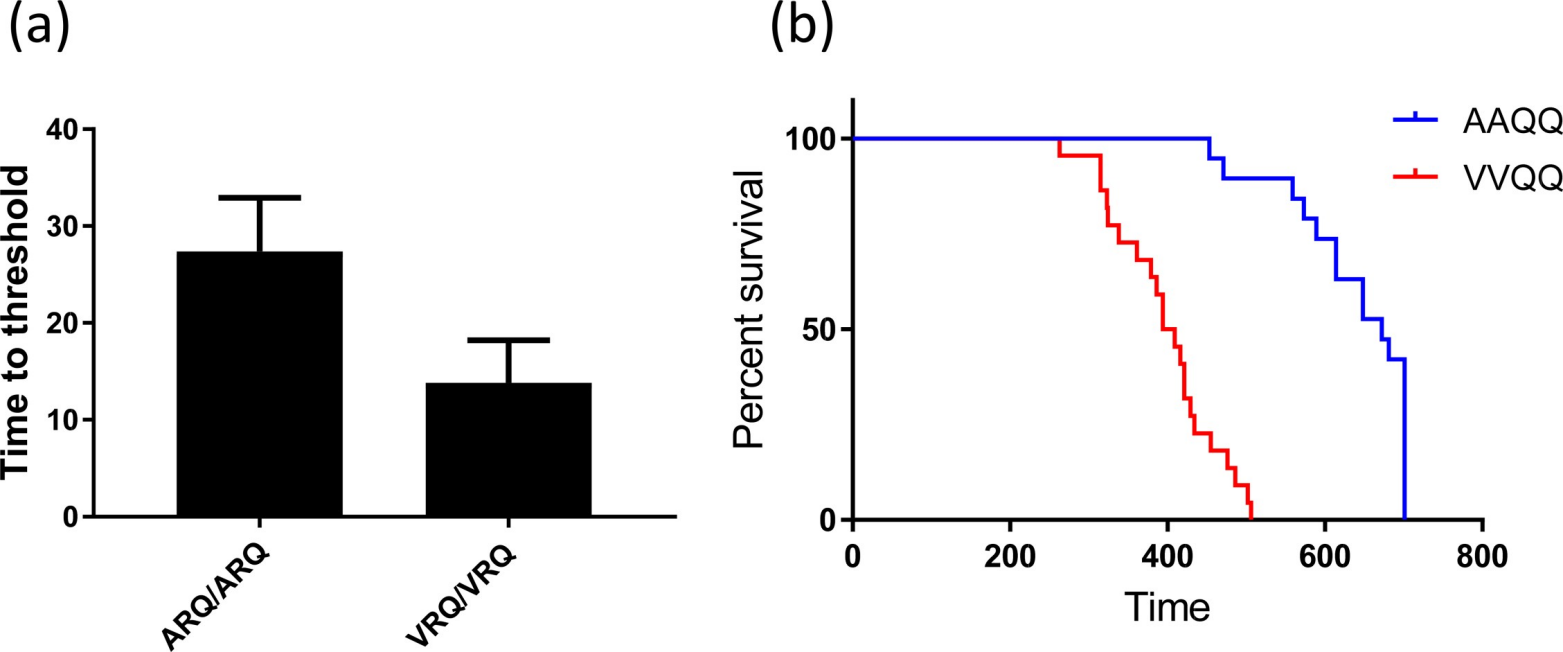
Comparison between average lag times obtained from RT-QuIC and incubation period from bioassay. A bar graph showing the mean and standard deviation for the average lag time for RT-QuIC reactions seeded with VRQ/VRQ and ARQ/ARQ. (b) Survival curve shows that Tg12 mice (132M) inoculated with a 1% brain homogenate of the scrapie inoculum from VRQ/VRQ sheep have a shorter mean incubation period than mice receiving inoculum from ARQ/ARQ sheep.

To evaluate the influence that *PRNP* genotype of the sheep scrapie donor had on the seeded conversion of elk PrP, RT-QuIC reactions containing recombinant elk proteins, 132L (Fig 6A) or 132M (Fig 6B), were seeded with 10^−2^ dilutions of EIA normalized brain tissues from two ARQ/ARQ sheep (#1 and #2) infected with x124 inoculum, two VRQ/VRQ sheep (#3 and #4) infected with x124 inoculum, two VRQ/VRQ sheep (#5 and #6) infected with No. 13–7 inoculum, and two ARQ/ARQ sheep (#7 and #8) infected with No. 13–7 inoculum. Assays utilizing elk substrate 132M showed fibril formation as monitored by ThT fluorescence to be earliest for x124 inoculated VRQ/VRQ sheep (#3 and #4) followed by No. 13–7 inoculated VRQ/VRQ sheep (#5 and #6) then No. 13–7 or x124 inoculated ARQ/ARQ sheep (#2, #7, and #8), all within 25 hours. Only assay seeded with x124 ARQ/ARQ sheep (#1) brain homogenate did not produce the positive ThT fluorescence (Fig 6B). Excluding negative control samples and assay seeded with brain sample #1, all other assays regardless of isolates or genotypes showed fluorescence within 25 hours. Interestingly, all assays using 132M substrate seeded with samples of either x124 or No. 13–7 scrapie from VRQ/VRQ sheep showed rapid increases in ThT fluorescence before 20 h, while the lag time of assays seeded with samples from ARQ/ARQ sheep inoculated with either x124 or No. 13–7 was approximately 25 hours. Assays with 132L substrate did not readily differentiate the genotypes for either isolate, however, assays seeded with brains from animal #3 and #4 (Fig 6A) inoculated with x124 showed the shortest lag time of any substrate, genotype, and isolate combination. This result indicates that the scrapie agent from sheep with the VRQ/VRQ genotype is more rapidly detected than equivalent samples derived from sheep with the ARQ/ARQ genotype. Difference in lag times between VRQ/VRQ and ARQ/ARQ seeds are shown in Fig 7A with VRQ/VRQ exhibiting a lag time of 13.8 ± 4.4 hours and ARQ/ARQ showing a lag time of 27.38 ± 5.5 hours. This further supports that scrapie samples from sheep of different *PRNP* genotypes can be distinguished by comparing seeding activities and biophysical properties of PrP^Sc^ after RT-QuIC using recombinant 132M elk prion protein.

### Mouse bioassay

In addition to *in vitro* assessment by RT-QuIC, we inoculated Tg12 mice (expressing 132M elk *PRNP*) with brain homogenates from sheep with clinical signs of scrapie. The inoculum genotypes were chosen to be consistent with relevant genotypes of sheep scrapie agent that could be present naturally and to mimic the upper and lower conversion efficiencies by RT-QuIC. More specifically, ARQ/ARQ sheep were not susceptible to x124 by the intranasal route while VRQ/VRQ x124 and ARQ/ARQ No. 13–7 reflect the shortest and longest RT-QuIC lag time. Mice inoculated with x124 from a VRQ/VRQ sheep (24/24; 100%) had difficulty moving, developed poor motor coordination, and were euthanized with a mean incubation period of 398 days post inoculation. Mice inoculated with No. 13–7 from an ARQ/ARQ sheep had a lower attack rate (20/25; 80%) with an average incubation period of 635 days. Many of the mice (8/25), however, tested positive at the end of the experiment (700 days post-inoculation) without developing clinical signs (Fig 7B).

## Discussion

The first description of chronic wasting disease in deer was in a captive population, but the original source of infection has never been determined [40]. Previous experimental studies demonstrated that elk are susceptible to the scrapie agent and develop clinical signs and lesions that are similar to chronic wasting disease by microscopic examination and immunohistochemical analysis [31, 41]. The sheep scrapie agent transmitted to three elk after intracerebral inoculation and those elk were shown to have PrP^Sc^ in the central nervous system [31]. The sheep scrapie agent was also transmitted to white-tailed deer by intracerebral inoculation [42, 43]. Another study showed that scrapie prions could be transmitted to cervids by using transgenic mice expressing elk *PRNP* (Tg12) [44]. In these earlier studies, the recipient genotype has been considered with regard to host susceptibility/resistance, yet for a variety of reasons inoculum genotype was not considered. Investigation of how scrapie inoculum from different genotypes of sheep may affect the transmission to cervid hosts with differing prion protein polymorphisms may help us to further understand the risks of transmission of the scrapie agent to cervids and may ultimately prove informative with regard to understanding the origin of CWD. To test how scrapie isolates derived from different genotypes of sheep can affect cervids, we performed RT-QuIC experiments by seeding recombinant elk prion proteins with two different strains of the scrapie agent from either ARQ/ARQ or VRQ/VRQ genotypes of sheep. We also characterized the effect of polymorphisms occurring in elk *PRNP* sequence, 132L and 132M, when seeded with samples of the scrapie agent from sheep of these two *PRNP* genotypes. Each substrate showed different seeding activity with brain samples in a genotype dependent manner. We show here that PrP^Sc^ from VRQ/VRQ sheep can seed the elk substrate (132M) with higher conversion efficiency than seed from ARQ/ARQ sheep. Also, we inoculated mice expressing elk prion protein (Tg12) with two scrapie isolates, x124 (VRQ/VRQ) and No. 13–7 (ARQ/ARQ). While this is a more limited set of host and recipient genotypes, the mouse bioassay supports the RT-QuIC data by showing a shorter incubation period for Tg12 mice inoculated with the scrapie agent from VRQ/VRQ sheep relative to the incubation period for Tg12 mice inoculated with the scrapie agent from ARQ/ARQ sheep.

The host *PRNP* genotype influences a host’s potential susceptibility to prion agents. For example, the relative susceptibility of sheep to the scrapie agent is influenced by polymorphisms in the amino acid composition of prion protein at residue 136 (valine or alanine), 154 (arginine or histidine) and 171 (glutamine or lysine). Sheep with the ARR/ARR genotype are resistant to scrapie, whereas sheep with VRQ/VRQ are highly susceptible [20, 45]. Previous studies describe two scrapie isolates (x124 [18] and No. 13–7 [46]) that exhibited differences in incubation periods after intracerebral inoculation. In the case of x124, the incubation period is affected in a pronounced genotype dependent manner. Sheep of the VRQ/VRQ or VRQ/ARQ genotypes inoculated with the x124 agent have incubation periods of less than 6 months, while sheep with the ARQ/ARQ genotype have incubation periods of at greater than 16 months [18]. In contrast, sheep infected with the No. 13–7 agent exhibited somewhat extended incubation periods for sheep with the VRQ/VRQ genotypes relative to sheep with ARQ/ARQ and that the PrP^Sc^ deposition profile was different between ARQ/ARQ sheep and VRQ/VRQ sheep infected with No. 13–7 indicating that different recipient genotypes influence the tissue distribution of scrapie [46].

The difference in fibril stability between VRQ/VRQ and ARQ/ARQ sheep inoculated with x124 scrapie is consistent with either the “cloud” hypothesis of prion strain switching [47, 48] or deformed templating [49] as it is possible that different strains are being selected for or generated in the ARQ/ARQ sheep than in the VRQ/VRQ sheep used as seeds in this study. However, this does not change the interpretation of the data presented here, that the genotype of the infectious material used can influence the apparent susceptibility in new host species because the observation of a seed genotype dependence is not exclusive to x124. The strain No. 13–7 does have a genotype dependence in the RT-QuIC conversion of elk 132M despite the absence of any stability difference that would support distinct strains [50].

The present study indicates that RT-QuIC reactions conducted in elk rPrP substrates that are seeded with PrP^Sc^ from different genotypes of sheep have different conversion efficiencies. For 132M elk substrate this is true for both scrapie isolates evaluated while for 132L elk substrate the PrP^Sc^ seed genotype differences are only observed with strain x124. As previously discussed, this is consistent with either cloud hypothesis or deformed templating in the x124 strain in different genotypes sheep by RT-QuIC. The distinct seeding activities from different genotypes of sheep PrP^Sc^ were also observed in our mouse bioassays. While a more limited selection of genotypes was assessed, the results of bioassays in transgenic mice expressing elk *PRNP* (132M) are consistent with the *in vitro* assay showing that mice infected with the x124 agent from sheep with the VRQ/VRQ genotype have shorter incubation periods than mice infected with the No. 13–7 agent from sheep with the ARQ/ARQ genotype. These differences in the incubation periods for transgenic mouse bioassays are also consistent with the incubation periods observed in the sheep inoculated with x124 inoculum and No. 13–7 [19]. It is worth noting that sheep with ARQ/ARQ genotype were resistant to infection with the x124 agent by the oronasal route indicating that x124 is unlikely to occur naturally in sheep with ARQ/ARQ genotype [19] making this source of inoculum irrelevant from the standpoint of transmission to elk. While the genotype dependence in sheep of x124 scrapie could indicate a strain switching event, the results of the current study using RT-QuIC still demonstrate that the genotype of the donor inoculum should be considered when assessing transmissibility, in particular those studies involving cross species transmission.

Cross species transmission of TSEs is a major concern to animal and human health. Considerable knowledge in this area has been gained utilizing both natural host species and transgenic animals [26, 31, 41, 42, 44]. The impact of the recipient genotype on TSE progress was the focus of these previously published studies and in the past little or no consideration was made with regard to the genotype of the inoculum source animals. This was a reasonable decision at the time based upon the status of TSE knowledge at the time and based on the numerous host-recipient genotype combinations that are possible being prohibitive for large animal and even transgenic rodent model studies. However, based upon the results presented here we must consider that studies of cross species transmission based solely on the recipient genotype provide a less than complete assessment of transmission risk. Advances in *in vitro* conversion based detection methods such as RT-QuIC offer the ability to determine whether the seed genotype influences risk of disease transmission across various combinations of both donor and recipient genotype.

## Conclusions

In this study, we addressed the effect of donor genotype with regard to the potential transmission of the sheep scrapie agent to elk by using *in vitro* conversion through RT-QuIC and compared these results to *in vivo* transmission experiments. Conversion efficiency with seed derived from the brain of a VRQ/VRQ sheep is greater than that observed for seed derived from an ARQ/ARQ sheep brain. Mouse bioassay also demonstrates a shorter incubation period and higher attack rates in transgenic mice expressing elk PrP when inocula is derived from VRQ/VRQ sheep. When conducting prion transmission studies, the genotype of the prion agent inoculum donor should be given careful consideration.
